# Sixth Annual Report of the Directors and Superintendent of the Ohio Lunatic Asylum to the Forty-third General Assembly, December 9, 1844

**Published:** 1845-04

**Authors:** 


1. *Sixth Annual Report of the Directors and Superintendent of the Ohio Lunatic Asylum to the Forty-third General Assembly*, December 9, 1844. pp. 67.

